# What quantitative mechanical loading stimulates *in vitro* cultivation best?

**DOI:** 10.1186/s40634-015-0029-x

**Published:** 2015-06-19

**Authors:** Jerry Natenstedt, Aimee C Kok, Jenny Dankelman, Gabrielle JM Tuijthof

**Affiliations:** Department of Biomechanical Engineering, Faculty of Mechanical, Materials and Maritime Engineering, Delft University of Technology, Mekelweg 2, Delft, 2628 CD The Netherlands; Department of Orthopedic Surgery, Academic Medical Centre, Meibergdreef 9, Amsterdam, AZ 1105 The Netherlands

**Keywords:** Chondrocytes, BMMSC, Compression, Mechanical loading, *in vitro*, Collagen type II, GAG, Cell therapy

## Abstract

Articular cartilage has limited regeneration capacities. One of the factors that appear to affect the *in vitro* cultivation of articular cartilage is mechanical stimulation. So far, no combination of parameters has been identified that offers the best results. The goal is to review the literature in search of the best available set of quantitative mechanical stimuli that lead to optimal *in vitro* cultivation.

The databases Scopus and PubMed were used to survey the literature, and strict in- and exclusion criteria were applied regarding the presence of quantitative data. The review was performed by studying the type of loading (hydrostatic compression or direct compression), the loading magnitude, the frequency and the loading regime (duration of the loading) in comparison to quantitative evidence of cartilage quality response (cellular, signaling and mechanical).

Thirty-three studies met all criteria of which 8 studied human, 20 bovine, 2 equine, 1 ovine, 1 porcine and 1 canine cells using four different types of cultivated constructs. Six studies investigated loading magnitude within the same setup, three studies the frequency, and seven the loading regime. Nine studies presented mechanical tissue response. The studies suggest that a certain threshold exits for enhanced cartilage *in vitro* cultivation of explants (>20 % strain and 0.5 Hz), and that chondrocyte-seeded cultivated constructs show best results when loaded with physiological mechanical stimuli. That is a loading pressure between 5–10 MPa and a loading frequency of 1 Hz exerted at intermittent intervals for a period of a week or longer. Critical aspects remain to be answered for translation into *in vivo* therapies.

## Introduction

**Fig. 1:**
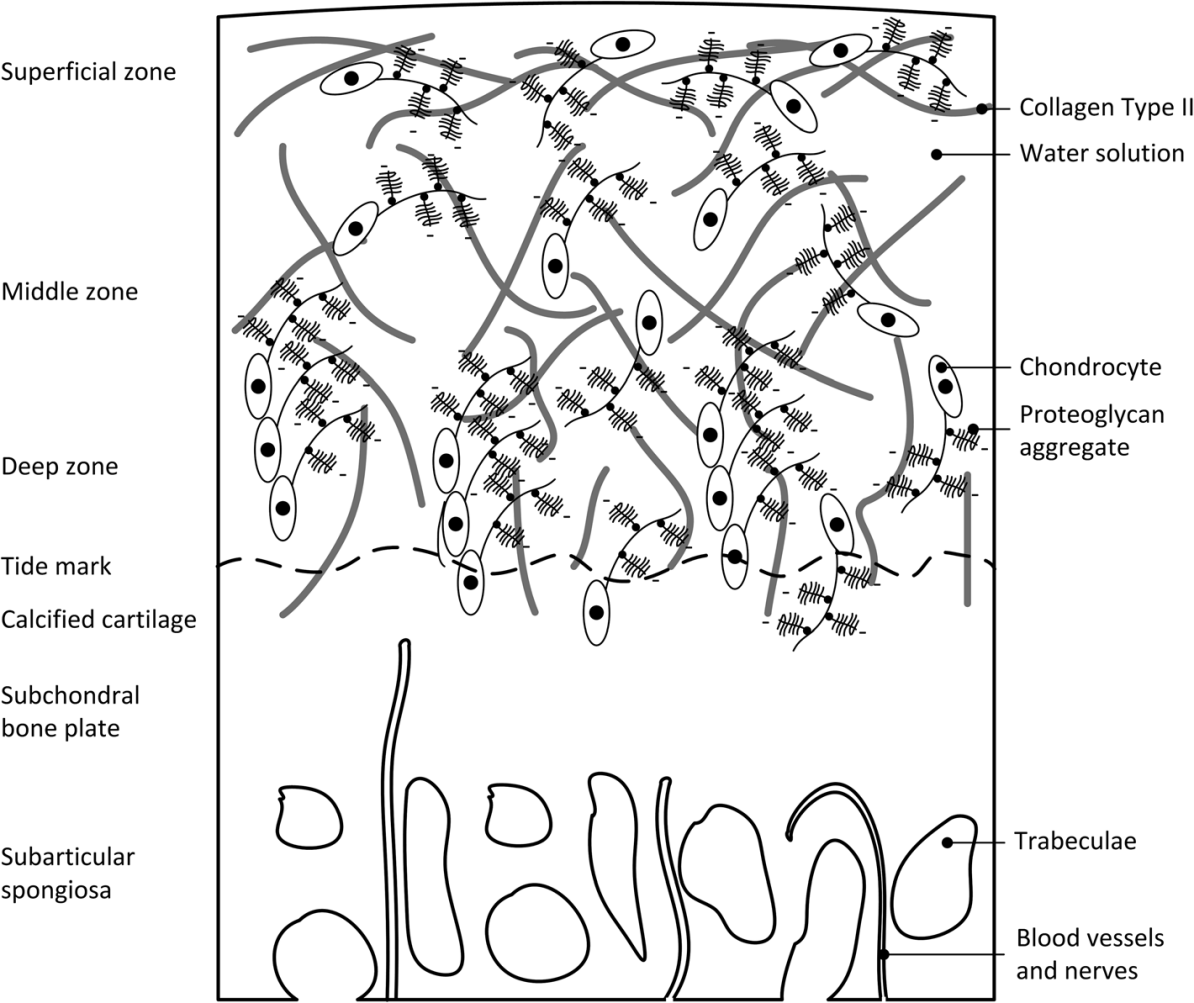
Cellular structure of cartilage. Defects are sustained in different layers: partial thickness chondral defect (up till the deep zone), full thickness chondral defect (up till the calcified cartilage) and osteochondral defect (crossing the subchondral bone plate) base on (Madry et al. 2010)

Articular cartilage is a nonlinearly permeable, viscoelastic multiphasic material containing chondrocytes and proteoglycan aggregates (3-10 % of volume) that are surrounded by an extracellular matrix (ECM), whose primary constituents are water with mobile ions (60-85 % of volume) and collagen type II (10-30 % of volume) (Fig. 1) (Mow et al. 1999; Schulz and Bader 2007; Khan and Scott 2009; Madry et al. 2010). Damaged articular cartilage presents itself as partial chondral, full thickness chondral or osteochondral defects (Fig. 1). Partial or full thickness lesions show limited ability to regenerate due to its avascular and highly structured nature, which prevents progenitor cells and chondrocytes to migrate to the defect-site (Heath and Magari 1996; Zengerink et al. 2006; Khan et al. 2008; Magnussen et al. 2008; Khan and Scott 2009). For osteochondral defects, the subchondral bone plate is breached leading to an inflow of blood containing bone marrow-derived mesenchymal stem cells (BMMSCs) that populate the defect site (Khan and Scott 2009; Madry et al. 2011). These cells may differentiate into chondrocytic cells, which in turn can regenerate the ECM (Angele et al. 2003; Bahuleyan et al. 2009). This repair tissue mostly contains collagen type I and degrades over time (Khan et al. 2008; Madry et al. 2011; Hannon et al. 2014). However, newer tissue engineered techniques are clinically applied in which tissue engineered constructs with or without (autologous) cells are used to enhance cartilage regeneration with more hyaline like cartilage as result (Brittberg 2010; Fortier et al. 2011; Hildner et al. 2011; Spiller et al. 2011). Over the last decade, numerous studies have been published that unravelled important factors influencing the cartilage regeneration process (e.g. (Chung and Burdick 2008; Khan et al. 2008; Brittberg 2010; Fortier et al. 2011; Hildner et al. 2011; Spiller et al. 2011)).

This review will focus on the mechanical loading to promote cartilage cultivation. Mechanical stimuli promote ECM production (Grodzinsky et al. 2000), increase chondrocyte activity, and aid to protect the ECM temporarily from excessive loading (Mow et al. 1999). Due to its unique composition, cartilage can be loaded up to 18 MPa *in vivo*, which is up to 15 times the body weight (Darling and Athanasiou 2003; Elder and Athanasiou 2009; Spiller et al. 2011). If an underdeveloped ECM sustains such high loading, it can collapse; and can further deteriorate eventually leading to a full stop in intrinsic repair (Darling and Athanasiou 2003). A similar mechanism is seen when damaged cartilage (with a disrupted ECM) is loaded during gait with physiological values of around 5 times the body weight (van Dijk et al. 2010a, 2010b).

On the other hand, biomechanical intermittent cyclic loading has shown to stimulate regeneration of cartilage tissue (Arokoski et al. 2000; Bonassar et al. 2001; Waldman et al. 2004; Chung and Burdick 2008; Fan and Waldman 2010; Hess et al. 2010; Potier et al. 2010). Tissue engineering studies show that dynamic compression increases cartilage *in vitro* cultivation rather than static compression (Schulz and Bader 2007; Elder and Athanasiou 2008, 2009; Mizuno and Ogawa 2011). Important loading parameters appear to be the magnitude, frequency and duration (Ikenoue et al. 2003). So far, no combination of parameters has been identified that offers the best result in *in vivo* regeneration. Unfortunately, a first search in the literature indicated that none of the retrieved *in vivo* studies provided quantitative values to identify this combination. Therefore, the goal of this study is to review the literature in search of the best available set of quantitative mechanical stimuli that increase cartilage *in vitro* cultivation; and possibly deduct suggestions for *in vivo* cartilage regeneration.

## Review

### Methods

The databases Scopus and PubMed where used to survey the literature. The following keywords and synonyms were used to retrieve candidate studies: (Mechan* OR Biomech*) AND (load* OR loading OR stimulat* OR compress* OR shear OR forces) AND articular cartilage AND (repair OR regeneration OR healing). Both original and review papers were included from 1980 until April 18th 2015 and the search was limited to English language. The in- and exclusion criteria were formulated based on strict interpretation of the research question.

#### Inclusion criteria

Studies were included if they:

- Studied dynamic loading.
- Harvested either chondrocyte or BMMSC cultures from larger animal models (canine, bovine, equine, porcine, human). Larger animals provide a closer environment and metabolism to the human cartilage case (Chu et al. 2010).
- Provided quantitative values of the applied mechanical stimuli (loading magnitude, frequency, type and regime).
- Provided quantitative values of the effect on the cartilage quality response (e.g. cellular or mRNA response in terms of increased percentage).

#### Exclusion criteria

Studies were excluded if they only:

- Used computational methods to simulate the mechanical properties or regenerating capabilities of cartilage.
- Determined the mechanical properties of cartilage (e.g. stiffness or elastic strain limit).
- Presented operative techniques to promote cartilage healing (e.g. grafting and graft-ingrowth, arthroplasty, or microfracturing).
- Studied *in vivo* loading to stimulate cartilage, for example by specific physical therapy protocols, and did not provide quantitative data on the mechanical stimuli and/or effect on the cartilage quality response.
- Studied non-articular cartilage (e.g. cartilage of the ear).
- Examined the effect of non-mechanical factors (e.g. hormonal, chemical, or electrical) that can stimulate cartilage cultivation or regeneration.
- Harvested chondrocytes or stem cells from small animals (e.g. rodent) (Chu et al. 2010).
- Performed continuous static compression as loading regime.

From the included studies, the cell donor and culture, the construct, the additives, the type of loading (hydrostatic compression or direct compression), the loading magnitude, the frequency and the loading regime (duration of the loading) were documented as input parameters, and the quantitative evidence of cartilage quality response (cellular, signaling and mechanical responses) was documented as output parameter. Unfortunately, variety in testing conditions such as cell source, cultivated constructs, and the chosen cartilage quality response parameters only allowed for a qualitative comparison. To enhance comparison, the applied loading regime is presented in one format: the frequency, the total time of loading per day, the total number of days and total loading hours. Additionally, the loads were mostly expressed in pressure (*P* or σ equals force per area expressed in Pa) (Shepherd and Seedhom 1999; Darling and Athanasiou 2003). However, some studies use the strain (*ℇ*), defined as the percentage of cartilage thickness decrease. To enhance uniformity, the strain values were converted into pressure values using the formula for linear elastic materials:

1 $$ \sigma =E\cdot \varepsilon $$

Where *E* is the Young’s modulus, which is a material property, and σ is the compression load expressed in Pa. Since cartilage is a viscoelastic multiphasic material (Mow et al. 1999), multiple parameters are needed to describe its material behaviour. In this study, an approximation of the Young’s modulus was used: the ‘instantaneous’ compressive modulus of cartilage (*E*_*c*_) (Shepherd and Seedhom 1999). Substituting *E*_*c*_ and *ℇ* in Equation (1) gives the compression load. The value of *E*_*c*_ depends on the joint donor site (Shepherd and Seedhom 1999). To this end, the range of values for the human knee joint (between 6 and 12 MPa) was filled out in Eq. (1) together with the applied strain to calculate *E*_*c*_ for studies that used the human knee, the bovine and canine stifle joint as donor sites (Shepherd and Seedhom 1999)*.* In a similar fashion, the range of values for the human ankle (between 11 and 19 MPa) was filled out for bovine or canine metatarsal donor joints (Shepherd and Seedhom 1999).

Finally, this review presents the changes found in cellular, signaling and mechanical response due to the mechanical stimuli, which were indicated as an increased (+) or decreased (−) response compared to controls or as no change or similar (=) (Tables 1, 2, 3, 4, 5).

**Table 1:** Results on changes in cellular, signaling and/or mechanical response to explants for hydrostatic and direct compression (Fig. 2). PG is proteoglycan; MMP is matrix metalloproteinases; # is number; h is hours; h/day is hours per day; − is decrease or inhibition; = is no change or status quo; + is increase; ++ is highest increase. *-symbol implies pressure converted from strain, which is added in brackets

Study	Cell source, cultivated construct, additive(s)	Magnitude (MPa)	Freq (Hz)	Loading (h/day)	Loading (# days)	Loading (total h)	Culture composition (change -, =, +)	mRNA response (change -, =, +)	Other findings (change -, =, +)
Hydrostatic compression									
Parkinnen 1993 (Parkkinen et al. 1993)	Bovine	5	0.5	1.5	1	1.5	+ PG synthesis		
	Explant		0.25						
	Fetal calf serum		0.05				= PG synthesis		
			0.0167						
Direct compression									
Li 2013 (Li et al. 2013)	Young Bovine Bruised Explant Serum free medium, 20 g/ml ascorbic acid	0.6-1.2* (10 %)	0.5	4	4	16	+ PG synthesis	+ aggrecan	Control show better results compared to bruised explants
								+ collagen II	
		1.2-2.4* (20 %)					++ PG synthesis	++ aggrecan	
								++ collagen II	
		1.8-3.6* (30 %)					+ PG synthesis	+ aggrecan	
								= collagen II	
Okuda 2013 (Okuda et al. 2013)	Young Bovine, Explant 20 % Fetal bovine serum; 50 mg/L L-ascorbic acid	0.6-1.2* (10 %)	1	3.5	5	17.5	+ sGAG		+ compressive modulus
							+ # of cells		
Torzilli 1996 (Torzilli et al. 1996)	Bovine, Explant 10 % Fetal bovine serum; 50 μg/mL ascorbic acid	1	1	24	1	24	- PG synthesis		
Torzilli 2011 (Torzilli et al. 2011)	Bovine, Explant 10 % Fetal bovine serum; 50 μg/mL ascorbic acid	0.5 (10 %)	0.5	24	3	72	= PG content	= MMP −3, −13

**Table 2:** Results on changes in cellular, signaling and/or mechanical response to chondrocyte-seeded meshes for hydrostatic and direct compression (Fig. 2). PGA is polyglycolic acid; PEGT/PBT is polyethylene glycol terephthalate/polybutylene terephthalate; Sox9 is the gene that regulates chondrogenic differentiation; # is number; h is hours; h/day is hours per day; − is decrease or inhibition; = is no change or status quo; + is increase; ++ is highest increase. *-symbol implies pressure converted from strain, which is added in brackets; **loading was performed every other day for 1 h twice a day with 8 h rest in between

Study	Cell source, cultivated construct, additive(s)	Magnitude (MPa)	Freq (Hz)	Loading (h/day)	Loading (# days)	Loading (total h)	Culture composition (change -, =, +)	mRNA response (change -, =, +)	Other findings (change -, =, +)
Hydrostatic compression									
Carver 1999a (Carver and Heath 1999a)	Young equine Mesh nonwoven PGA 10 % Fetal bovine serum; 50 μg/mL ascorbic acid	3.4	0.25	2	35	70	+ GAG		GAG increase strongest at 6.9 MPa for young
							= collagen II		
							= # of chondrocytes		
		6.9					+ GAG		
							+ collagen II		
							= # of chondrocytes		
	Adult equine Mesh nonwoven PGA 10 % Fetal bovine serum; 50 μg/mL ascorbic acid	3.4					+ GAG		Collagen II increase strongest at 6.9 MPa for young and adult
							+ collagen II		
							= # of chondrocytes		
		6.9					= GAG		
							+ collagen II		
							= # of chondrocytes		
Carver 1999b (Carver and Heath 1999b)	Young equine Mesh nonwoven PGA 10 % Fetal bovine serum; 50 μg/mL ascorbic acid	3.44	0.25	2	35	70	+ GAG		+ E- modulus
							+ collagen		
							= # of chondrocytes		
Direct compression									
Démarteau 2003 (Démarteau et al. 2003)	Human Mesh PEGT/PBT Foam 10 % Fetal bovine serum; growth factor TGF-β1, FGF-2, PDGFbb	0.3-0.6* (5 %)	0.1	4	3	12	+ GAG	= Sox9	Measured peak loading 0.018 MPa
								= aggrecan	
								= collagen II	
Hilz 2014 (Hilz et al. 2014)	Bovine, Mesh Polyurethane 25 % Fetal calf serum;50 μg/mL L-ascorbic acid	1.2-2.4* (20 %)	1	2**	21	16	+ GAG	=Sox9	
							+ aggrecan	+collagen II	
							+ collagen II		
El-ayoubi 2011 (El-Ayoubi et al. 2011)	Canine, Mesh poly-L-Lactide 10 % Fetal bovine serum	0.6-1.2* (10 %)	1	3	14	42	+ # of cells

**Table 3:** Results on changes in cellular, signaling and/or mechanical response to chondrocyte cultivated constructs for hydrostatic compression (Fig. 2). PG is proteoglycan; MMP is matrix metalloproteinases; h is hours; h/day is hours per day;;- is decrease or inhibition; + is increase; ++ is highest increase; = is no change or status quo. *-symbol implies pressure converted from strain, which is added in brackets, **-symbol is increased aggrecan only with 4 h

Study	Cell source, cultivated construct, additive(s)	Magnitude (MPa)	Freq (Hz)	Loading (h/day)	Loading (# days)	Loading (total h)	Culture composition (change -, =, +)	mRNA response (change -, =, +)	Other findings (change -, =, +)
Ikenoue 2003 **(Ikenoue et al.** 2003 **)**	Human Monolayer 10 % Fetal bovine serum	1	1	4	1	4		= aggrecan	Loading of 16h gave better results compared to 4h
								= collagen II	
		5	1					+ aggrecan	
		10						= collagen II	
		1	1	4	4	16		+ aggrecan	
								+ collagen II	
		5	1					+ aggrecan	
								++ collagen II	
		10	1					++ aggrecan	
								++ collagen II	
Elder 2008 **(Elder and Athanasiou** 2008 **)**	Young bovine, Agarose gel, 20 % Fetal bovine serum; 50 μg/mL L-ascorbic acid	1	0.1	1	5	5	+ GAG		= Aggregate modulus
							= collagen II and # of cells		= E-modulus
		5	0.1				= GAG		+ Aggregate modulus
							= collagen II and # of cells		= E-modulus
		10	0.1				+ GAG		= Aggregate modulus
							= collagen II and # of cells		++ E-modulus
		1	1				= GAG		+ Aggregate modulus
							= collagen II and # of cells		+ E-modulus
		5	1				= GAG		= Aggregate modulus
							= collagen II and # of cells		= E-modulus
		10	1				++ GAG		++ Aggregate modulus
							= collagen II and # of cells		++ E-modulus
Hu 2006 **(Hu and Athanasiou** 2006 **)**	Young bovine, Agarose gel, 10 % Fetal bovine serum; 50 μg/mL L-ascorbic acid	10	1	4	40	160	= GAG (no loss) + collagen II		= Aggregate modulus
Mizuno 2011 **(Mizuno and Ogawa** 2011 **)**	Young bovine, Aggregate pellet, Collagen solution, 10 % Fetal bovine serum	0.5	0.5	24	7	168	+ sGAG	+ aggrecan, + collagen II, + MMP-3, −13	
Kawanishi 2007 **(Kawanishi et al.** 2007 **)**	Young bovine, Aggregate pellet, 10 % Fetal bovine serum; 50μg/mL ascorbic acid	5	0.5	4	4	16	+ GAG	+ aggrecan	
							+ sGAG	+ collagen II	
Suh 1999 **(Suh et al.** 1999 **)**	Bovine, Monolayer, 10 % Fetal bovine serum	−0.08 vacuum	0.14	6	1	6	+ PG synthesis	+ aggrecan	
							= collagen synthesis	= collagen II	
Parkinnen 1993 **(Parkkinen et al.** 1993 **)**	Bovine, Monolayer, 10 % Fetal calf serum	5	0.5	1.5	1	1.5	- PG synthesis		
			0.25						
			0.05						
			0.0167				= PG synthesis		
			0.5	20	1	20	+ PG synthesis		
			0.25						
			0.05				= PG synthesis		
			0.0167				- PG synthesis		
			0.0082				= PG synthesis		
			0.0034						
Jortikka 2000 **(Jortikka et al.** 2000 **)**	Bovine, Monolayer, 10 % Fetal bovine serum	5	0.5	20	1	20	+ PG synthesis		
Smith 1996 **(Smith et al.** 1996 **)**	Bovine, Monolayer Ham’s F-12 medium; 3 % Fetal bovine serum	10	1	4	1	4	+ PG synthesis	+ aggrecan	
								+ collagen II	
Smith 2000 **(Smith et al.** 2000 **)**	Bovine, Monolayer, Ham’s F-12 medium	10	1	2,4,8,12, 24	1	2,4,8,12, 24		+ aggrecan**, + collagen II	Superior increase compared to one loading period
				4	4	16		++ aggrecan, ++ collagen II	
Heyland 2006 **(Heyland et al.** 2006 **)**	Porcine chondrocytes, Beads alginate, 10 % Fetal bovine serum	0.3	0.0083	6	4	24	+ GAG, = collagen II		
				6	7	42	= GAG, + collagen II		= E-modulus

**Table 4:** Results on changes in cellular, signaling and/or mechanical response to chondrocyte cultivated constructs for direct compression (Fig. 2). OA is osteoarthritis; MMP is matrix metalloproteinases; PG is proteoglycan; h is hours; h/day is hours per day; − is decrease or inhibition; + is increase; ++ is highest increase; = is no change or status quo. *-symbol implies pressure converted from strain, which is added in brackets,** -symbol is increase only present 12 h post stimulation, ^-symbol is increase only present after 6 h post stimulation

Study	Cell source, cultivated construct, additive(s)	Magnitude (MPa)	Freq (Hz)	Loading (h/day)	Loading (# days)	Loading (total h)	Culture composition (change -, =, +)	mRNA response (change -, =, +)	Other findings (change -, =, +)
Nebelung 2012 (Nebelung et al. 2012)	Human OA Hydrogel collagen type I 10 % Human serum	0.6-1.2* (10 %)	0.3	24	28	672	= proteoglycan	= aggrecan	= E-modulus
							= collagen II	+ collagen II	
								+ MMP-13	
Shelton 2003 (Shelton et al. 2003)	Bovine, Agarose gel Type VII 20 % Fetal calf serum	1.7-2.9* (15 %)	0.3	24	2	48	- GAG		
			1				+ GAG		
			3				= GAG		
Omata 2012 (Omata et al. 2012)	Bovine, Agarose gel Type VII 20 % Fetal bovine serum; 0.85 mM L-ascorbic acid	1.7-2.9* (15 %)	1	6	22	132			+ E-modulus
Hung 2004 (Hung et al. 2004)	Bovine, Agarose gel Type VII 10 % Fetal bovine serum; growth factor: TGF-β1, IGF-1	0.6-1.2* (10 %)	1	3	3	9		+ aggrecan	= aggregate modulus
				3	20	60			+ E-modulus
									+ aggregate modulus
Nicodemus 2010 (Nicodemus and Bryant 2010)	Young bovine, Hydrogel polyethylene glycol, 5 % Fetal bovine serum; 50 mg/L L-ascorbic acid	1.2-2.4* (20 %)	0.3	24	7	168	+ GAG	+ aggrecan	
								- collagen II	
								- MMP-3	
								= MMP-13	
				6	7	42	= GAG	= aggrecan	
								+ collagen II	
								+ MMP-3, −13	
Waldman 2004 (Waldman et al. 2004)	Bovine, Monolayer on top of calcium polyphosphate mesh, 5 % Fetal bovine serum	0.3-0.6* (5 %)	1	.1 (400 cycles)	3.5	0.5	= PG synthesis		
							++ coll. synthesis		
		0.6-1.2* (10 %)					++ PG synthesis		
		1.2-2.4* (20 %)					= coll. synthesis		
		0.3-0.6* (5 %)		0.6 (2000 cycles)	3.5	2	= PG synthesis		
							+ coll. synthesis		
		0.6-1.2* (10 %)					+ PG synthesis		
		1.2-2.4* (20 %)					= coll. synthesis		
		0.3-0.6* (5 %)	1	0.1	7	1	= PG synthesis		= E-modulus
							= coll. synthesis		
				0.1	14	2	+ PG synthesis		+ E-modulus
							+ coll. synthesis		
De Croos 2006 (De Croos et al. 2006)	Bovine, Monolayer on top of calcium polyphosphate mesh 5 % Fetal bovine serum	0.001	1	<1 h	1	<1 h	+ PG synthesis ^	+ aggrecan **	
							+ coll. synthesis ^	+ collagen II **	
								+ MMP-3, −13

**Table 5:** Results on changes in cellular and/or signaling response to BMMSC cultivated constructs under hydrostatic compression (Fig. 2). Sox9 is the gene that regulates chondrogenic differentiation; h is hours; h/day is hours per day; − is decrease or inhibition; and + is increase; ++ is highest increase; = is no change or status quo

Study	Cell source, cultivated construct, additive(s)	Magnitude (MPa)	Freq (Hz)	Loading (h/day)	Loading (# days)	Loading (total h)	Culture composition (change -, =, +)	mRNA response (change -, =, +)
Mesh								
Wagner 2008 (Wagner et al. 2008)	Human BMMSC, Mesh Collagen Type 1 50 mg/mL bovine serum albumin; 50 μg/mL L-ascorbic acid; 10^−9^ M dexamethasone	1	1	4	10	40	+ proteoglycan	+ Sox9
								+ aggrecan
								+ collagen II
Luo 2007 (Luo and Seedhom 2007)	Ovine BMMSC, Mesh non-woven filamentous plasma-treated polyester 10 % Fetal bovine serum; 50 μg/mL ascorbic acid; 10^−7^ M dexamethasone	0.1	0.25	0.5	7	3.5	+ GAG	
							= collagen	
				0.5	10	5	++ GAG	
							+ collagen	
Gel								
Miyanishi 2006a (Miyanishi et al. 2006a)	Human BMMSC, Aggregate pellet 1.25 mg/mL bovine serum albumin; 50 μg/mL ascorbic acid; 10^−7^ M dexamethasone	0.1	1	4	14	56	= sGAG	+ Sox9
								+ aggrecan
								= collagen II
		1					+ sGAG	++ Sox9
								+ aggrecan
								= collagen II
		10					++ sGAG	++ Sox9
								++ aggrecan
								+ collagen II
Miyanishi 2006b (Miyanishi et al. 2006b)	Human BMMSC, Aggregate pellet 1.25 mg/mL bovine serum albumin; 50 cpg/mL ascorbic acid; 10^−7^ M dexamethasone	10	1	4	14	56		+ Sox9
								+ aggrecan
								+ collagen II
Angele 2003 (Angele et al. 2003)	Human BMMSC, Aggregate pellet 10 % Fetal bovine serum	5.03	1	4	1	4	= proteoglycan	
							= collagen	
				4	7	28	+ proteoglycan	
							+ collagen	
Finger 2007 (Finger et al. 2007)	Human BMMSC, Agarose gel Type VII Growth medium Cambrex	7.5	1	4	14	56		= Sox9

### Results

After a first title and abstract scan of the 836 hits combined from Scopus and PubMed, 106 papers were left to be reviewed completely, which eventually resulted in 33 studies that were eligible for inclusion. Generally, the tissue engineering studies investigated the effect of mechanical stimuli on cultivated constructs at least in one study arm compared to unstimulated controls (Darling and Athanasiou 2003; Mauck et al. 2003). Tables 1, 2, 3, 4, 5 summarize the results.

#### Cell sources, cultivated constructs and additives

Two cell sources (chondrocytes and BMMSC) were studied from 6 mammal types: 8 studies used human cells, 20 bovine, 2 equine, 1 ovine, 1 porcine and 1 canine. First, 27 studies used chondrocytes as cell source (Tables 1, 2, 3, 4). Chondrocytes are responsible for the production of the ECM (Meachim and Stockwell 1973; Buckwalter and Mankin 1998; Cohen et al. 1998), since they are likely to synthesize collagen type II and proteoglycans (Schulz and Bader 2007; Spiller et al. 2011). Four different constructs were used to culture chondrocytes: a) explants, which consist of a complete section of cartilage that is excised from a cadaver and embedded in a culture medium (Parkkinen et al. 1993) (Table 1), b) tissue engineered meshes that have a structural 3D shape (Table 2), c) monolayers that consist of isolated chondrocytes from full thickness pieces of cartilage seeded onto a plate (Jortikka et al. 2000; Smith et al. 2000) (Tables 3–4), d) hydrogels that have a softer structure compared to meshes (Carver and Heath 1999b; Hu and Athanasiou 2006) (Tables 3–4). One study used serum free medium (Li et al. 2013), one study added human serum (Nebelung et al. 2012), three added calf serum, and twenty one studies added bovine serum, with thirteen studies also adding L-ascorbic acid and two also adding growth factors in conjunction (Tables 1, 2, 3, 4).

Second, BMMSCs were harvested from bone marrow, and centrifuged to become a pellet culture (Miyanishi et al. 2006a; Kawanishi et al. 2007) (Table 5). Two cultivated constructs were used onto which BMMSCs were seeded : a) a gel or pellet composition and b) tissue engineered meshes (Luo and Seedhom 2007; Wagner et al. 2008) (Table 5). Bovine serum was added in five out of 6 studies, with four studies also adding 50 μg/mL L-ascorbic acid and dexamethasone.

### Loading regime

**Fig. 2:**
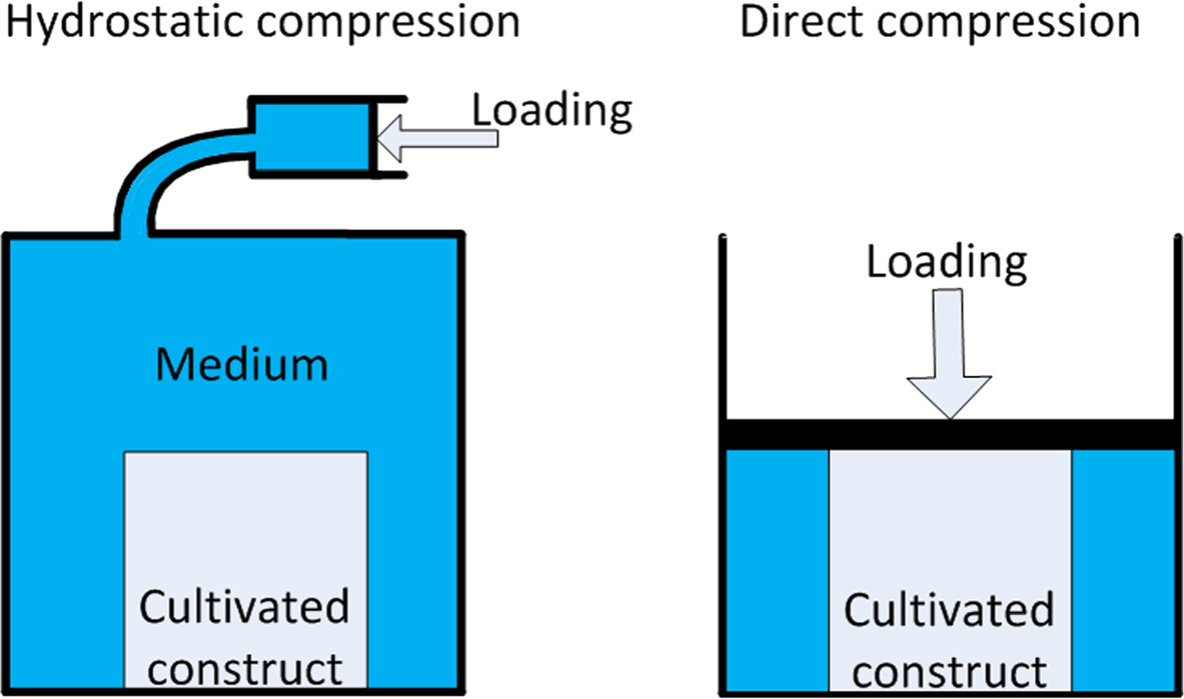
Difference between hydrostatic compression (left) and direct compression (right). The arrows indicate the loading direction

Two types of cyclic compression were applied: hydrostatic or direct compression (Fig. 2). Hydrostatic pressure is applied by compressing the fluid surrounding the tested culture with a piston (Elder and Athanasiou 2009) (Tables 1, 2, 3 and 5). Direct compression implies that a piston directly presses on the tissue, which is commonly expressed in percentage of strain (Demarteau et al. 2003) (Tables 1,2 and 4). Except the studies by Torzelli et al. (Torzilli et al. 1996; Torzilli et al. 2011) and De Croos et al. (De Croos et al. 2006), all other eleven studies required conversion from strain to pressure (Tables 1, 2 and 4).

The loading magnitude, frequency and regime varied highly. For example Carver and Heath stimulated their samples with 6.9 MPa at 0.25Hz for 2 h per day over a period of 35 days (Carver and Heath 1999b), while Démarteau et al. applied a loading of 5 % of strain with 0.1Hz for 4 h per day over a period of 3 days (Démarteau et al. 2003). In contrast to this, five out of six studies with human derived BMMSC used 1 Hz as loading frequency for 4 h per day (Table 5) (Angele et al. 2003; Miyanishi et al. 2006a, 2006b; Finger et al. 2007; Wagner et al. 2008).

### Cartilage cellular, signaling and mechanical response parameters

Three types of methods were found to document cartilage quality response: cellular, signaling, and mechanical responses (Tables 1, 2, 3, 4, 5). Cellular response is routinely determined with histology, which allows identification of specific (macro) molecules that typically represent healthy cartilage (proteoglycans, glucosaminoglycans (GAGs) and sulfated glucosaminoglycans (sGAGs), collagen type II). An increase of proteoglycans is typically determined using staining with Safranin O (Darling and Athanasiou 2003; Schulz and Bader 2007). Similarly, the increase of GAGs and sGAGs is determined with dimethylmethylene blue assay staining (Farndale et al. 1986; Carver and Heath 1999b; Shelton et al. 2003; Heyland et al. 2006; Hilz et al. 2014). The increase in collagens is determined by staining with Picrosirius red, Masson’s trichrome stain, or antibody-staining such as anti-collagen antibodies or monoclonal antibodies, and/or the use of polarized light (Angele et al. 2003; Darling and Athanasiou 2003; Heyland et al. 2006; Elder and Athanasiou 2008; Nicodemus and Bryant 2010). After staining, the histologic samples are further interpreted with histologic scores and compared to control samples to indicate relative cellular responses. The type of collagen is assessed with immunohistochemistry (Elder and Athanasiou 2008). Finally, cellular response in the form of proteoglycan synthesis is routinely determined by assessing the radioactive labeled ^35^Sulfate-uptake by the proteoglycans (Parkkinen et al. 1993; Torzilli et al. 2011; Li et al. 2013).

Signaling response indirectly indicates the potential of the cells to (de)differentiate into cartilage, because it assesses changes in the level of mRNA expression as produced by the chondrocyte cells with a reverse transcription polymerase chain reaction (RT-PCR) (Darling and Athanasiou 2003; Schulz and Bader 2007). A change in proteoglycan production is commonly documented as an increase in aggrecan mRNA that is the core protein backbone to which GAGs chains are attached (Tables 1, 2, 3, 4) (Démarteau et al. 2003; Ikenoue et al. 2003; Schulz and Bader 2007). With the same method other relevant expressions are assessed: collagen type II, the gene sex determining region box 9 (Sox9) and Matrix metalloproteinases (MMP). Sox9 is indicative for the regulation of chondrogenic differentiation and plays a role in expression of collagen type II and aggrecan (Démarteau et al. 2003; Miyanishi et al. 2006a; Finger et al. 2007; Wagner et al. 2008; Hilz et al. 2014). MMP plays a major role in ECM turnover and degradation (Hilz et al. 2014). MMP-3 has shown to be a key player in degrading matrix and inactivating other degrading enzymes (Cawston and Wilson 2006; Echtermeyer et al. 2009), and MMP-13 seems to influence the progress of osteoarthritis (Hilz et al. 2014).

Mechanical responses were documented by compression tests of the samples and determining the Young’s (or E-) modulus from the linear range of the stress–strain curve (Carver and Heath 1999b; Hung et al. 2004; Heyland et al. 2006; Hu and Athanasiou 2006; Elder and Athanasiou 2008; Omata et al. 2012). Cartilage quality response was given as change of cellular response (including synthesis) by 27 of 33 studies, as change of signaling response by 21 of 33 studies, and as change of mechanical response by 9 of 33 studies.

#### Responses to mechanical stimuli

Due to the difference in cell sources, cultivated constructs, compression and outcome measures only qualitative comparison could be performed. Bovine explant testing mimics the *in vivo* case most closely especially the study by Li et al. (Li et al. 2013), since they used bruised explants (Table 1). The studied variations in the loading regime of the explants suggest a certain threshold for the magnitude of loading (>10 % or > 1.2 MPa) and the frequency (>0.5 Hz) to stimulate increased proteoglycan synthesis (Table 1). Only Okuda et al. (Okuda et al. 2013) confirmed that this was correlated to increased mechanical response. Studies that tested equine chondrocyte-seeded meshes by hydrostatic compression support the need for a loading threshold (Table 2), since increased cell density was observed by higher loading magnitudes and correlated to increased mechanical response (Carver and Heath 1999b). The three studies of chondrocyte-seeded meshes by direct compression presented too much variation to point in a specific loading regime (Table 2).

The studies that use monolayer and gel cultivated constructs and test variations in loading magnitude, frequency or loading regime (Tables 3–4) also suggest the need for thresholds in magnitude and frequency with a trend towards higher values (up to 5–10 MPa and up to 1 Hz) compared to the explant studies to achieve increased responses (Parkkinen et al. 1993; Smith et al. 2000; Ikenoue et al. 2003; Shelton et al. 2003; Waldman et al. 2004; Elder and Athanasiou 2008). Tables 3–4 also highlight the effect of different loading regimes, which seems to suggest that prolonged duration and loading at intervals (no continuous intermittent loading) increase cell density and synthesis, signaling response as well as mechanical response (e.g. (Shelton et al. 2003; Hung et al. 2004)). Exceptions are the studies by De Croos (De Croos et al. 2006) who find increased response at a low magnitude of 0.001 MPa applied for less than 1 h, and by Hu and Athanasiou (Hu and Athanasiou 2006) who did not find a change in the E-modulus after prolonged loading for 160 h.

The studies using BMMSCs as cell source support the suggestion that the largest increase in cellular and signaling response is achieved for larger loading magnitudes (>5 MPa) at a frequency of 1 Hz for a prolonged period (>7 days) at intermittent intervals (Table 5). However, no mechanical responses were measured for these constructs.

### Discussion

Five studies mimicked the *in vivo* case most closely by testing bovine explants. Only two of these varied the loading magnitude or frequency, which suggest the need of a certain threshold (>20 % strain and > 0.5 Hz) for increased proteoglycan synthesis (Table 1). A careful qualitative interpretation of the results suggests that for chondrocyte-seeded cultivated constructs a loading pressure between 5–10 MPa and a loading frequency of 1 Hz exerted at intermittent intervals for a period of a week or longer are recommended as appropriate mechanical stimulus. These values are in the physiologic range of normal gait (Waters et al. 1988; Giddings et al. 2000; Brand 2005; Doke et al. 2005; van Dijk et al. 2010a). Due to the variety of testing conditions and methods to express cartilage quality response, only qualitative comparison was possible, which poses limitations to the study. First, differences in sample tissue, sample preparation, donor type and donor age all accounted for differences in the outcome of these studies (Parkkinen et al. 1993; Carver and Heath 1999a; Darling and Athanasiou 2003; Chung and Burdick 2008). Still, fourteen of the 33 studies did measure the cartilage quality response to varying loading parameters within the same set up. Even though, signaling response does not always reflect actual cellular and mechanical changes, the studies that report them also report cellular and/or mechanical response in conjunction (Tables 2, 3, 4, 5), with in the majority of the cases showing corresponding in- or decreases. With this, the decision was made to include studies that only present signaling response (four in total), since two have varied the loading regime (intermittent vs continuous loading (Smith et al. 2000); and loading magnitude and duration (Ikenoue et al. 2003)) as needed to answer our main research question (Table 3). Second, two different types of compression were applied: hydrostatic (Tables 1, 2, 3 and 5) and direct compression (Tables 1,2 and 4). There is an on-going debate which type of loading is more physiological. Some are in favor of direct compression (Buschmann et al. 1995; Mauck et al. 2000; Waldman et al. 2004). Also, direct compression allows continuous measurement of mechanical responses, but needs some tricks to be applied to soft constructs by placing the samples in bags (De Croos et al. 2006). Bachrach et al. (Bachrach et al. 1998) suggest that hydrostatic pressure seems to be more representative for the *in vivo* loading case, because it mimics the viscoelastic multiphasic cartilage behavior closest. A drawback is that it also stimulates the sides of the samples. An advantage of applying hydrostatic compression is that it allows for harmonization of the applied load, and it allows mechanical stimulation of different types of cultivated constructs including the softer ones. The proposed transformation procedure from strain to pressure seems to make sense, because the calculated pressure values are in line with the values found in other studies: 3.6 MPa leads to a 29 % strain (Herberhold et al. 1999) vs 20 % strain (Hilz et al. 2014) (Tables 2 and 4). However, it still remains an approximation, which needs to be interpreted with care. Third, biologic demonstration of the increase in cartilage quality response is highly important, since it indicates parameters (signals, cells types, cell synthesis) that should be triggered to stimulate the cell activity and behave like cartilage. However, documentation of actual mechanical response would be expected as well, since this determines performance. In one quarter of the studies (9 out of 33) the mechanical response was measured, which is a rather low percentage. Some of the cultivated constructs (monolayer, pellet) do not resemble the actual ECM structure, which makes mechanical testing difficult or impossible (Tables 1, 2, 3, 4, 5). Full characterization is difficult, because of its highly complex viscoelastic behavior (Mow et al. 1999; Schulz and Bader 2007; Khan and Scott 2009; Madry et al. 2010). Furthermore, constructs can also change due to the loading or do not necessarily mimic mechanical cartilage behavior (Nebelung et al. 2011). This latter is supported by conflicting results that were found for two studies in which agarose gel was used: Hu and Athanasiou (Hu and Athanasiou 2006) show that a 20 % increase in collagen type II does not seem to influence the mechanical properties (Table 3), and others (Hung et al. 2004; Elder and Athanasiou 2008; Omata et al. 2012) did not find a relation between histologic and mechanical parameters.

*In vivo* tissue engineering cartilage repair techniques (e.g. Matrix-Induced Autologous Chondrocyte Implantation or cell-seeded hydrogel plugs (Brittberg 2010; Fortier et al. 2011; Hildner et al. 2011; Spiller et al. 2011)) make use of similar scaffolds. This review gives a summary of current evidence, which can be used for future development of on *in vivo* application rehabilitation protocol. Several factors are fundamentally different for the *in vivo* case, including the fact that the ECM is not intact as result of the cartilage lesion, and that the access to essential biologicals (e.g. cytokines, growth factors) is different in the physiologic situation compared to the *in vitro* situation. Especially, the boundary between the healthy cartilage and tissue engineered scaffold is a vulnerable spot (Khan et al. 2008), which most likely cannot withstand the suggested loading magnitude (Guettler et al. 2004; Khan et al. 2008; van Dijk et al. 2010a, 2010b; Spiller et al. 2011; Hunt et al. 2012). However, the results could be used to optimize preconditioning of tissue engineered scaffolds before implantation into patients (Shelton et al. 2003; Nebelung et al. 2012; Omata et al. 2012). Therefore, the timing of loading could be a critical factor that needs to be further explored. For example the testing period might be even further extended (Waldman et al. 2004), since *in vivo* studies with animal models evaluated the cartilage quality response after long testing periods (56 days or longer), much longer than those found in this review (Saris et al. 2003; Kok et al. 2013; Miller et al. 2014; Ortved et al. 2015). Finally, studying the dynamic compression of damaged explants (e.g. (Li et al. 2013)), should be elaborated to identify the best magnitude, frequency and loading regime, since these constructs mimic the *in vivo* cartilage lesion closest. This will facilitate the translation of the found combination of mechanical parameters to patients.

### Conclusions

Overall, the results seem to suggest that a certain threshold exits for enhanced cartilage *in vitro* cultivation of explants, and that chondrocyte-seeded cultivated constructs show best results when loaded with physiological mechanical stimuli. This seems a reasonable conclusion, because nature is highly optimized for daily activities such as normal walking. Critical aspects remain to be answered for translation of the results into *in vivo* therapies.
